# Macrophages Modulate the Function of MSC- and iPSC-Derived Fibroblasts in the Presence of Polyethylene Particles

**DOI:** 10.3390/ijms222312837

**Published:** 2021-11-27

**Authors:** Qi Gao, Zhong Li, Claire Rhee, Shiqi Xiang, Masahiro Maruyama, Elijah Ejun Huang, Zhenyu Yao, Bruce A. Bunnell, Rocky S. Tuan, Hang Lin, Michael S. Gold, Stuart B. Goodman

**Affiliations:** 1 Orthopaedic Research Laboratories, Department of Orthopaedic Surgery, Stanford University School of Medicine, Stanford, CA 94304, USA; qigao7@stanford.edu (Q.G.); cr21@stanford.edu (C.R.); masa1460@stanford.edu (M.M.); eehuang@stanford.edu (E.E.H.); zhenyuy@stanford.edu (Z.Y.); 2 Center for Cellular and Molecular Engineering, Department of Orthopaedic Surgery, University of Pittsburgh School of Medicine, Pittsburgh, PA 15219, USA; alanzhongli@pitt.edu (Z.L.); xiangs@pitt.edu (S.X.); tuanr@cuhk.edu.hk (R.S.T.); hal46@pitt.edu (H.L.); 3 Department of Microbiology, Immunology and Genetics, University of North Texas Health Science Center, Fort Worth, TX 76107, USA; Bruce.Bunnell@unthsc.edu; 4 Institute for Tissue Engineering and Regenerative Medicine, The Chinese University of Hong Kong, Hong Kong, China; 5 Department of Neurobiology, University of Pittsburgh School of Medicine, Pittsburgh, PA 15219, USA; msg22@pitt.edu

**Keywords:** fibrosis, micro physiological system (MPS), synovial lining

## Abstract

Fibroblasts in the synovial membrane secrete molecules essential to forming the extracellular matrix (ECM) and supporting joint homeostasis. While evidence suggests that fibroblasts contribute to the response to joint injury, the outcomes appear to be patient-specific and dependent on interactions between resident immune cells, particularly macrophages (Mφs). On the other hand, the response of Mφs to injury depends on their functional phenotype. The goal of these studies was to further explore these issues in an in vitro 3D microtissue model that simulates a pathophysiological disease-specific microenvironment. Two sources of fibroblasts were used to assess patient-specific influences: mesenchymal stem cell (MSC)- and induced pluripotent stem cell (iPSC)-derived fibroblasts. These were co-cultured with either M1 or M2 Mφs, and the cultures were challenged with polyethylene particles coated with lipopolysaccharide (cPE) to model wear debris generated from total joint arthroplasties. Our results indicated that the fibroblast response to cPE was dependent on the source of the fibroblasts and the presence of M1 or M2 Mφs: the fibroblast response as measured by gene expression changes was amplified by the presence of M2 Mφs. These results demonstrate that the immune system modulates the function of fibroblasts; furthermore, different sources of differentiated fibroblasts may lead to divergent results. Overall, our research suggests that M2 Mφs may be a critical target for the clinical treatment of cPE induced fibrosis.

## 1. Introduction

Although total joint arthroplasty (TJA) is a highly successful surgical procedure, approximately 3–10% of individuals develop chronic inflammation and fibrosis of the synovial membrane post-TJA [1]. The hallmark of fibrosis includes aberrant deposition of collagen and expression of α-smooth muscle actin (α-SMA) and immune cell infiltration [2,3]. These biological processes alter the normal biochemical composition and functional state of the stroma. Accordingly, fibrosis and chronic inflammation of the synovium after joint replacement reduces joint motion and adversely affects quality of life [4].

The synovial lining of the knee joint undergoes a series of well-described changes in the presence of chronic inflammatory conditions characterized by hypertrophic synovitis [5], neovascularization and recurrent pain [6]. These changes are in marked contrast to the phenotype of a healthy synovial membrane composed of a layer of fibroblasts and Mφs, which produce the extracellular matrix (ECM) that maintains the structural framework and homeostasis of the joint [7]. Wear debris particles and other byproducts generated from joint arthroplasties are key drivers of synovial fibrosis [8]. These particles are thought to create an inflammatory response by activating Mφs that phagocytose wear debris generated from the joint arthroplasty [9,10]. However, there is considerable heterogeneity between joint arthroplasty patients concerning the extent of fibrosis observed. While this may reflect variability in the number of particles generated, it may also reflect differences in the Mφ response, specifically the balance between pro-inflammatory Mφs of the M1-phenotype and anti-inflammatory Mφs of the M2-phenotype [11]. Thus, an enhanced understanding of the biological mechanisms and interactions of fibroblast–immune cell interactions may yield substantial benefits to patients undergoing joint replacement surgery.

Mesenchymal stem cells (MSCs), such as those derived from bone marrow, share several features with fibroblasts, including surface markers, morphology, and potential immunophenotypic properties [12]. Fibroblasts differentiated from MSCs are widely used in tissue engineering studies, including synovial membranes and tendons [13]. In addition, enhanced collagen and tenascin expression are markers that are often used to confirm fibroblastic differentiation [14]. Another source of fibroblasts is human induced pluripotent stem cells (iPSCs), which not only enable the generation of a more significant number of fibroblasts than MSCs, but because they can be derived from patients with different clinical characteristics, they provide a robust approach to the modeling of human diseases and potential translational and clinical applications for personalized cell therapies [15,16,17]. Clinically relevant iPSC-derived constructs can be used for high throughput assays and in vitro disease models. Compared with established somatic cell lines, the experimental capabilities of unique iPSC lines may accelerate drug discovery and improve personalized precision medicines.

To explore the impact of wear particles on fibroblasts, the potential contribution of fibroblast specific properties on the response, as well as the relative contribution of M1- and M2- Mφs on the fibroblast response, we have developed and applied an in vitro model involving a co-culture of MSC- and iPSC-derived fibroblasts (MSC-Fs and iPSC-Fs) and Mφs with and without polyethylene particles. Co-cultures were grown in a 3D photocrosslinked methacrylated gelatin (GelMA) hydrogel to better mimic tissue architecture exposed to different clinical scenarios. We found that the presence of M2 phenotype Mφs increased functional fibroblast markers significantly. However, an opposite result was observed when M1 Mφs were co-cultured MSC-Fs and iPSC-Fs. There was also experimental variability between the iPSC-derived model and the MSC-derived model. These results suggest a potential modulatory effect of Mφs on fibroblasts in the inflammatory environment provided by wear particles and the importance of employing patient-specific cell-based models to assess clinical diagnosis and develop promising therapeutic strategies.

## 2. Results

### 2.1. Polyethylene Particles Induce the Expression of Functional Fibroblast Markers

**Figure 1:**
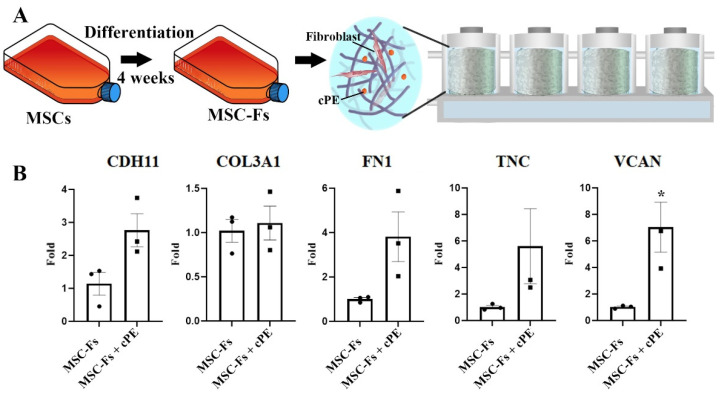
cPE induces the expression of functional fibroblast markers in MSC-Fs. (**A**) Experimental setup. MSC-Fs were seeded inside a 3D scaffold with or without polyethylene particles. (**B**) qPCR revealed an increased gene expression of fibroblast marker genes in MSC-Fs upon exposure to cPE, with a significant difference for *VCAN*. (* *p* < 0.05) (values are ±standard error of the mean (SEM)).

As a dominant cell type in the synovial membrane, fibroblasts play a vital role in regulating the ECM, producing collagen, and modulating synovial fluid lubricant and contractility [18]. The synovial fibroblasts are adversely affected by wear particles from joint replacements and can cause progressive loss of normal joint function. To investigate the impact of polyethylene debris on fibroblasts, we first differentiated MSCs to fibroblasts. After four weeks of incubation, the MSC-Fs were collected, and high expression of fibroblast marker genes, including fibronectin (*FN1*), versican (*VCAN*), collagen type III (*COL3A1*), cadherin (*CDH11*), and tenascin C (*TNC*), were detected. These fibroblasts were then cultured in a dynamic 3D environment, as shown in Figure 1A [19]. To mimic the in vivo situation, polyethylene particles (4.62 ± 3.76 µm [20]) coated with lipopolysaccharide (LPS) (cPE) were mixed with MSC-Fs. Fibroblasts with or without cPE were seeded in a 3D photo-crosslinked GelMA hydrogel scaffold. Four scaffolds were connected in series in one bioreactor. After another four weeks of culture, the expression of fibroblast markers was assessed. Relative to no particle controls, higher marker levels were observed in cultures with cPE; these differences were significant (*p* < 0.05) for VCAN (Figure 1B).

### 2.2. Mφ Accelerate Fibrosis in the Presence of Polyethylene Particles

**Figure 2:**
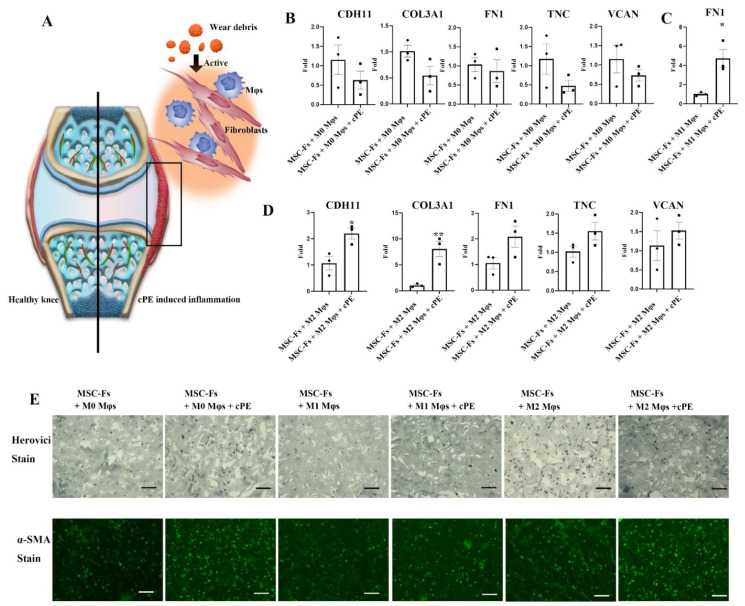
The effect of Mφs on the modulation of fibroblast functions in the presence of cPE. (**A**) Illustration of a synovial joint. Wear debris released after joint arthroplasty activates Mφs and fibroblasts in the synovial membrane. (**B**) Comparison of levels of fibroblast marker genes in the MSC-Fs and M0 Mφ co-culture system, (**C**) MSC-Fs and M1 Mφ co-culture system, and (**D**) MSC-Fs and M2 Mφ co-culture system, with and without the presence of cPE (* *p* < 0.05, ** *p* < 0.01) (values are ±SEM). (**E**) Herovici staining to visualize collagen and α-SMA immunofluorescence staining (scale bar, 100 μm).

cPE particles were found to active Mφs and created an inflammatory response, as shown in Figure 2A. To test the function of activated Mφs on fibroblasts in a cPE environment, freshly isolated monocytes were transformed to naïve Mφs, and the naïve Mφs (M0 Mφ) were polarized to pro-inflammatory (M1) Mφ or anti-inflammatory (M2) Mφ. These Mφs were then used in a co-culture of MSC-Fs with and without cPE in 3D scaffolds. Mφs from four donors were co-cultured to minimize donor-dependent heterogeneity. Mφs co-cultured with fibroblasts without cPE served as the control experiment, whereas the cPE treatment group served as the experimental group. A direct comparison of gene expression in the particle stimulation group vs. the particle-free co-culture group is shown in Figure 2B–D. When the fibroblasts were co-cultured with M0 macrophages with cPE, all the functional genes in fibroblasts were initially in the same range as in the M0 control group with no differences. Therefore, we further investigated the impact of activated Mφs on fibroblasts. In the M1 co-culture system, *FN1* increased significantly in the cPE experimental group. In addition, *CDH11*, *COL3A1*, *TNC*, and *VCAN* were robustly expressed in the cPE stimulated group but were undetectable in the particle-free control group. In the M2 co-culture system, marked increased gene expression levels of *CDH11* and *COL3A1* were observed in the cPE stimulated group.

Collagen content is one of the crucial factors to assess fibrotic progression [21]. Herovici staining was employed to determine the effect of cPE on collagen deposition. The sections showed dark blue staining in all samples, indicating immature collagen content, as shown in Figure 2E. However, more intense staining was observed in the cPE groups compared to the control groups, consistent with a cPE-induced increase in immature collagen. Fibroblasts transdifferentiate into myofibroblasts upon activation, which are identified as effector cells in the fibrogenesis process [22]. One critical marker in myofibroblasts is α-SMA, which is correlated with the fibrogenic process and contractile activities [23,24]. The immunofluorescent staining of α-SMA was consistent with a cPE-induced increase in the presence of myofibroblasts, likely resulting in higher tissue contractility and increased potential for tissue remodeling. There was no difference in either M1 Mφ or M2 Mφ co-culture system with or without cPE. The gene expression data were consistent with the immunostaining results, confirming that the level of fibrosis-related factors was elevated in the cPE stimulated group. These results showed that different Mφ phenotypes are not equally effective in inhibiting the fibrotic process when exposed to cPE.

**Figure 3:**
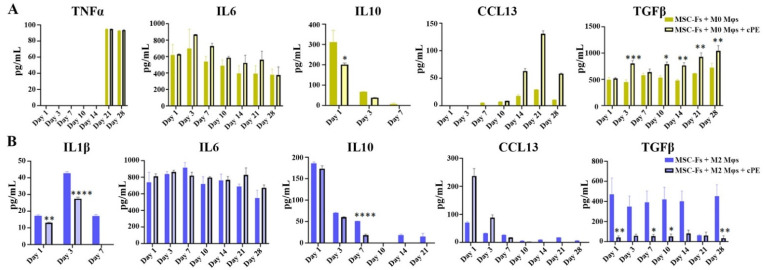
Concentration of cytokines in the perfusate from our fibroblast-Mφ co-culture systems with or without the presence of cPE. (**A**) M0 co-culture system. (**B**) M2 co-culture system. (* *p* < 0.05, ** *p* < 0.01, *** *p* < 0.001, **** *p* < 0.0001) (values are ±SEM).

Naïve (M0) Mφs respond to local stimulation and are polarized to either M1 Mφs, which enhance inflammation, or M2 Mφs that promote tissue regeneration. The impact of differentially polarized Mφs in the 3D co-culture was then studied. We measured the expression levels of cytokines, including interleukin 1β (IL-1β) 6 (IL-6), tumor necrosis factor (TNF) α, interleukin 10 (IL-10), transforming growth factor (TGF) β, C-C motif chemokine ligand 13 (CCL13), and C-C motif chemokine ligand 18 (CCL18) using enzyme-linked immunosorbent assay (ELISA) (Figure 3). We observed time-dependent changes in several cytokines and an influence of cPE on the time-dependent changes in cytokines, as shown in Figure 3A. The cytokine profile of the M0 control group revealed an increasing trend of CCL13 production, whereas the secretion of IL10 decreased and was not detectable after day 7. Collectively, these data suggested that M0 Mφs may polarize into functional Mφs over time. This polarization process appeared to be accelerated in the presence of cPE. For example, there was a higher level of TGFβ production in the cPE group. In addition, a higher level of CCL13 was observed in the cPE group on day 14, day 21, and day 28, consistent with the polarization of the M0 Mφs to an M2 phenotype. The cytokine levels of IL-6 and TNFα exhibited no significant change in the cPE group. In the M2 co-culture system, cPE stimulation significantly decreased the TGFβ level at all time points compared with the control cells, and a decrease in IL1β levels occurred during the first seven days, as shown in Figure 3B. An increase in CCL13 was observed in the cPE group during the first three days, but the levels decreased afterward.

Changes in cytokine levels were confirmed using qPCR. *CCL13*, *IL1β*, *IL6*, and *TNFα* were not detected in the M0 co-culture group but could be detected in the cPE experimental group. None of these genes was detected in the M1 co-culture system, the M1 control group, or the M1-cPE experimental group. In the M2 co-culture system, *CCL13*, *IL1β*, *IL6*, and *TNFα* were all elevated in the cPE group compared to the control group.

### 2.3. Cell Source-Specific Model for Polyethylene Particle Disease

**Figure 4:**
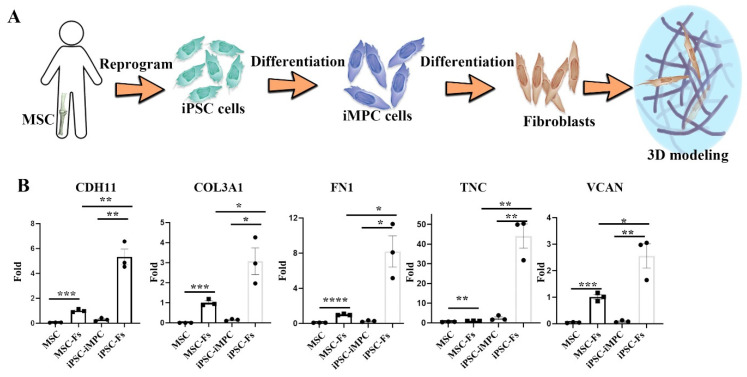
The effects of cPE on iPSC-Fs. (**A**) iPSC cells were differentiated to iMPCs first. Then, the iPSC-Fs were used to model the synovial membrane. (**B**) Expression of fibroblast-associated genes analyzed by qPCR (* *p* < 0.05, ** *p* < 0.01, *** *p* < 0.001, **** *p* < 0.0001) (values are ±SEM).

To determine whether the source of fibroblasts influences the response to cPE and Mφs, iPSCs were used to generate induced mesenchymal progenitor cells (iMPCs) [25,26], which were then differentiated into fibroblasts. Figure 4A illustrates this differentiation process. qPCR was performed to verify the differentiation process and compare it with MSC-Fs. The iPSC-Fs resembled MSC-Fs after 28 days of differentiation, robustly expressing *CDH11*, *COL3A1*, *FN1*, *TNC*, and *VCAN*, as shown in Figure 4B. In addition, these fibroblast-associated genes were also detected in MSC-Fs, but at lower levels. This result indicates that iPSC-derived, differentiated fibroblasts may be used in synovial modeling studies.

The iPSC-Fs were first challenged with cPE alone in the 3D bioreactor system. The constructs were cultured for 4 weeks, where the medium was perfused into the scaffold chamber. The influence of cPE on fibroblast gene expression was again quantified with qPCR. *TNC*, *FN1*, *COL3A1*, *CDH11* and *VCAN* were not detectable in the control group but were expressed in cPE treated group, indicating that cPE contributed to the potential risk of fibrosis.

### 2.4. M1 Mφs Inhibit Fibrosis in iPSC-Fs

**Figure 5:**
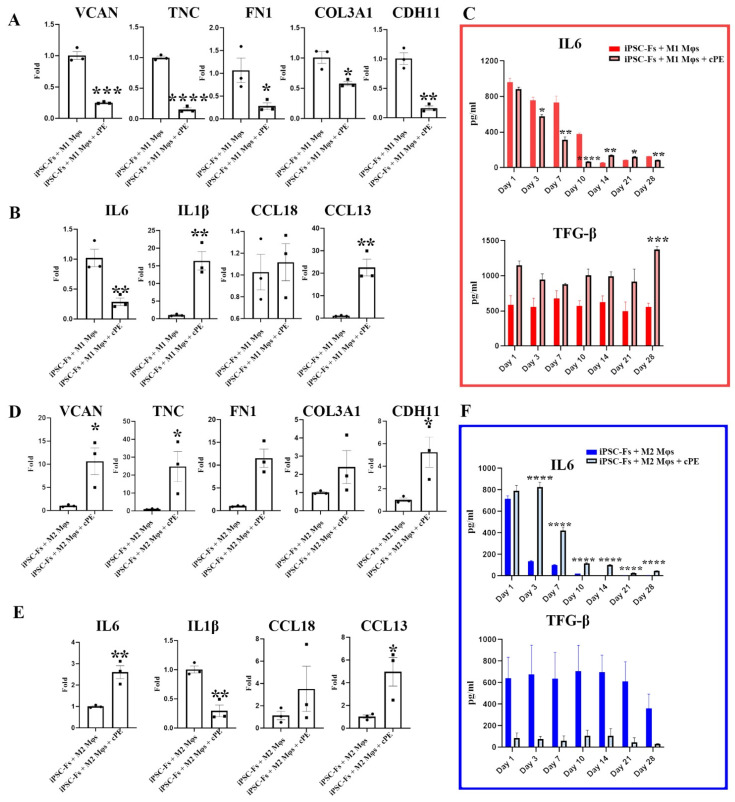
Impact of polyethylene particles on iPSC-Fs. Comparison of expression levels of (**A**) fibroblast-associated genes and (**B**) inflammation-related genes in the iPSC-Fs and M1 Mφ co-culture system. (**C**) Cytokine profile of the M1 co-culture system. Comparison of expression levels of (**D**) fibroblast-associated genes and (**E**) inflammation-related genes in iPSC-Fs and M2 Mφ co-culture system. (**F**) Cytokine profile of the M2 co-culture system. (* *p* < 0.05, ** *p* < 0.01, *** *p* < 0.001, **** *p* < 0.0001) (values are ±SEM).

We next assessed the impact of cPE on iPSC-Fs co-cultured with Mφs. These results are shown in Figure 5. Mφ phenotypes were defined via the expression of inflammatory and anti-inflammatory cytokines during the experiments. Gene expression levels were assessed using qPCR, and the cytokine profiles were determined via ELISA. Interestingly, changes in cytokine levels in iPSC-Fs were different from those observed in MSC-Fs. Specifically, when the iPSC-Fs were co-cultured with M1 Mφs, the expression levels of *FN1*, *VCAN*, *COL3A1*, *CDH11*, and *TNC* were decreased, even in the presence cPE, as shown in Figure 5A. In the presence of cPE, levels of *IL1β* and *CCL13* were elevated after four weeks of co-culture in the M1 Mφs group. However, there was no influence of cPE on *CCL18* levels in the M1 Mφs group. These results suggest that the impact of cPE Mφs polarization was mixed. That is, some M1 properties were enhanced (e.g., IL1β expression), but others were suppressed, as if at least some of the M1 Mφs had been switched to an M2 phenotype. By producing specific cytokines, M2 Mφs stimulate collagen production. In the M2 co-culture system, iPSC-Fs expressed higher *FN1*, *VCAN*, *COL3A1*, *CDH11*, and *TNC* in the cPE group than the control group, similar to the similarly co-cultured MSC-Fs. To further evaluate the phenotype of the Mφs that started with an M2 phenotype, we quantified inflammation-related gene expression. As with the M1 Mφs, the activity profile of the M2 Mφs appeared to be mixed. That is, the pro-inflammatory cytokines *IL1β* was suppressed in the presence of cPE, while the pro-inflammatory cytokine *IL6* and anti-inflammatory cytokine *CCL13* were elevated.

**Figure 6:**
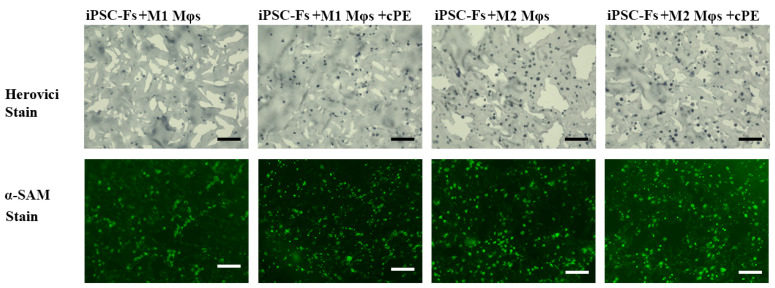
Herovici staining of collagen and immunofluorescent staining of α-SMA (scale bar, 100 μm).

Fibroblasts are known to contribute to the synthesis and remodeling of ECM in tissues. The balance of deposition and degradation of the ECM is crucial for maintaining tissue homeostasis. Thus, we also assessed the collagen content of the iPSC-Fs constructs using the Herovici staining. Collagen content was indicated by red staining for mature collagen and blue for immature collagen. Immature collagen was detected in all groups, as shown in Figure 6, with no detectable influence of cPE. There was no detectable influence of cPE on fibroblast remodeling as reflected by changes in α-SMA staining in the M1 group. However, α-SMA staining appeared to be elevated in the M2 groups with or without cPE.

## 3. Discussion

Fibroblasts in the synovial membrane secrete unique proteins that make up the ECM. FN1, VCAN, COL3A1, CDH11, and TNC are major elements in the ECM components and provide the ECM with structural integrity and support for cells [27,28,29]. Wear-generated particles lead to the accumulation of excess ECM components, resulting in fibrosis [30]. Prior research reported significant increases in collagen and fibronectin detected in patients after joint replacement [1]. Abnormal deposition of ECM results in pathological disease processes. For example, the increased accumulation of VCAN is reported in viral diseases. VCAN is an important part of the ECM that is also involved in tissue inflammation [14]. In response to damage associated with wear particles, fibroblasts produce soluble factors that recruit Mφs and regulate the immune response [31]. We were able to recapitulate these changes in our model system, where co-cultures of MSC-Fs fibroblasts with either M0, or M1, or M2 Mφs in the presence of cPE resulted in different expressions of fibroblast associated markers. Specifically, there was no statistical difference in M0 Mφ co-culture groups. In the M1 Mφ control group, the expression of most of the fibroblast function-related genes was at undetectable levels. However, cPE stimulation in the M1 Mφ coculture group triggered fibroblasts to express the functional markers *FN1*, *CDH11*, *COL3A1*, *TNC* and *VCAN*, while in the M2 Mφ coculture groups, cPE stimulation markedly increased the expression of *CDH11* and *COL3A1*, indicating increased activation of fibroblasts. Interestingly, MSC-Fs appeared to drive M0 Mφs toward an M2 phenotype, as cPE was associated with M2 cytokine production in cultures started with M0 Mφs. Furthermore, M2 Mφs-related cytokine production was enhanced in the M2 co-culture group.

In this study, we assessed the application of iPSCs to develop the joint disease model. iPSCs were used to generate iMPCs, which were further differentiated into fibroblasts. The iMPCs exhibited typical MSC characteristics, including morphology and surface marker profile [32]. In addition, the capabilities of osteogenic, chondrogenic, and adipogenic differentiation of iMPCs were comparable with those of MSCs. In the iPSC-Fs monoculture, the cPE stimulated group showed high expression levels of fibrosis-related genes. Given that iPSC-Fs expresses markers comparable to those detected in MSC-Fs, we tested the effects of Mφs on iPSC-Fs in the presence of polyethylene particles. A comparison of the normal group with the cPE stimulated group indicated M1 Mφs inhibit the risk of fibrosis in iPSC-Fs. These results suggest that patient-specific iPSCs are adaptable for joint disease modeling.

Mφs play pivotal roles in homeostasis, including the synovium of diarthrodial joints [33,34]. The number of Mφs increases dramatically during acute and chronic inflammation [35]. There is evidence of a bi-directional interaction between Mφs and fibroblasts. For example, as observed here, Mφs have been shown to regulate fibroblast function [36]. In contrast, fibroblasts have been shown to regulate the function of Mφs via cytokine secretion and the expression of cell surface markers [37]. Our results suggest that this bi-directional interaction is associated with changes in Mφs phenotype. For example, when Mφs were co-cultured with iPSC-Fs, cPE associated with increases in both the pro-inflammatory cytokine *IL1β* and anti-inflammatory cytokine *CCL13* was expressed when M1 Mφs were co-cultured with iPSC-Fs. The enhanced *CCL13* expression was consistent with a certain degree of Mφ repolarization. By comparison, after 4 weeks of incubation, a similar trend of *CCL13* was observed in the M2 co-culture system, but with a lower expression of *IL1β*. These results show that cPE stimulated the anti-inflammatory properties of M2 Mφs and induced the overexpression of the functional genes of fibroblasts, compared with the M2 control group. By comparison *CCL13* and *IL1β*, cPE aggravated proinflammation cytokines in M1 Mφs, but enhanced the anti-inflammation cytokines in M2 Mφs. Thus, while the M2 Mφs are known to exhibit the capacity for phagocytosis that mitigates inflammatory responses, they may also play a crucial role in inducing fibrosis [38]. For instance, M2 Mφs release TGF-β, which promotes fibroblast-to-myofibroblast differentiation and contributes to fibrogenesis [39]. Our results are consistent with the role of M2 Mφs in fibrosis and therefore indicate M2 Mφs might be a critical target for mitigating fibrosis.

Interestingly, there were marked differences between MSC-Fs and iPSC-Fs with respect to resting levels of gene expression, the response to cPE, and the interaction with Mφs. For example, cPE stimulation increased the function of MSC-Fs when co-cultured with M1 Mφs but not iPSC-Fs. This conflicting result needs further investigation as to the basis for the differences. Still, it is consistent with our hypothesis that there may be patient-specific factors that not only influence the properties of fibroblasts but also the bi-directional interaction between fibroblasts and immune cells. These differences may have important implications for treatment, and the model systems employed here may be leveraged to optimize potential interventions.

## 4. Materials and Methods

### 4.1. Cell Culture and Differentiation

Human bone marrow-derived MSCs were differentiated in fibrogenic medium (Advanced DMEM supplemented with 5% FBS, 1% GlutaMAX, 1% antibiotic-antimycotic, and 50 μg/mL Ascorbic acid) for four weeks. The culture medium was changed every seven days. All experiments were performed with passage 4–6 cells.

Mφs were isolated from de-identified male donors 20 to 40 years of age. The EasySep™ Human Monocyte Isolation Kit (Stem Cell Technologies) was employed to extract monocytes from the buffy coat. First, the cells were differentiated in Mφ stimulating medium (RPMI supplemented with 10% FBS, 5% antibiotic-antimycotic, 1% antibiotic-antimycotic, and 100 ng/mL Mφ colony-stimulating factor) for five days to obtain naïve Mφs (M0). Next, 20 ng/mL IFNγ and 10 ng/mL LPS were used to polarize the undifferentiated M0 Mφs to the M1 phenotype. Alternatively, 20 ng/mL IL-4 was added to obtain the M2 phenotype.

### 4.2. iPSC Culture and Differentiation

Induced mesenchymal progenitor cells (iMPCs) were generated from iPSCs using a spontaneous differentiation protocol as described before [26,32]. When iPSCs reached 70~80% confluency, the iPSCs’ maintenance medium was switched to STEMdiffTM-ACF Mesenchymal Induction Medium (Stemcell Technologies, Vancouver, BC, Canada) for three days, and the medium was changed every day afterwards. The medium was subsequently replaced by MesenCultTM-ACF Plus Medium (Stemcell Technologies) for four days of differentiation. The cells were detached and plated onto pre-coated plates within the MesenCultTM-ACF Plus medium. When the cultures reached 70~80% confluency, ACF Enzymatic Dissociation Solution and ACF Enzyme Inhibition Solution (Stemcell Technologies) were used for detachment; the differentiated cells were then grown in tissue culture flasks (Corning, NY, USA) for future experiments. The iMPCs were cultured in the fibroblast differentiation medium for four weeks as described above for MSCs before use.

### 4.3. Bioreactor Setup

GelMA was prepared following a previously developed protocol [39]. To enhance the inflammatory reaction stimulated by PE particles, we coated the particles, after ethanol sterilization, with 10 ng/mL LPS. Macrophages were then co-cultured with fibroblasts at a 2:1 ratio in a 3D photocrosslinked GelMA hydrogel scaffold with or without cPE. The final concentration of LPS and cPE in the scaffold were 0.25 ng/mL and 0.125%, respectively. Briefly, the cell-seeded hydrogels were photopolymerized using non-ultraviolet light (395 nm) within a custom-designed bioreactor, as described previously [19]. The constructs were cultured in the bioreactor for four weeks. The flow rate in the bioreactor was set to 2 μL/min. After four weeks of incubation, constructs were harvested using a 3-mm skin punch for analysis.

### 4.4. qPCR Analysis

mRNA was extracted from the constructs using Trizol reagent. After reverse transcription into cDNA with iScript Reverse Transcription Supermix for RT-qPCR (Bio-Rad, Hercules, CA, USA), qPCR was carried out to quantify gene expression. Taqman Gene Expression primers (Thermo Fisher Scientific, Waltham, MA, USA) were used, including tumor necrosis factor α (*TNFα*) (Hs00174128_m1), interleukin-1β (*IL-1β*) (Hs01555410_m1), interleukin 6 (*IL-6*) (Hs00174131_m1), C-C motif chemokine ligand 13 (*CCL13*) (Hs00234646_m1), C-C motif chemokine ligand 18 (*CCL18*) (Hs00268113_m1), versican (*VCAN*) (Hs00171642_m1), cadherin 11 (*CDH11*) (Hs00901479_m1), collagen type III α1 chain (*COL3A1*) (Hs00943809_m1), fibronectin 1 (*FN1*) (Hs01549976_m1), Tenascin-C (*TNC*) (Hs01115665_m1) and glyceraldehyde 3-phosphate dehydrogenase (*GAPDH*) (Hs02786624_g1). GAPDH was used as the housekeeping gene.

### 4.5. Enzyme-Linked Immunosorbent Assay (ELISA)

Cytokine secretion was analyzed by ELISA at different time points. ELISA kits of TNFα(50-112-8921), IL6 (88-7066-86), TGF-β(88-8350-86), and IL10 (88-7106-86) were obtained from Thermo Fisher Scientific, and the other kits, IL1β(DY201), CCL13(DY327), CCL18(DY394) were purchased from R&D Systems (R&D systems, Minneapolis, MN, USA). Briefly, the capture antibody was immobilized to the surface of a microplate, and then the plate was blocked to cover the unsaturated binding sites. After adding standards and samples, the nonspecific molecules were washed and removed to reduce the background. Next, a detection antibody was used to immobilize and quantify the target proteins. Streptavidin-HRP substrate and TMB were used as chromogenic reagents. Finally, A_450_ values were measured spectrophotometrically using a microplate reader.

### 4.6. Histology and Imaging

The constructs were collected and fixed with buffered 4% paraformaldehyde (PFA) overnight. After equilibration in 15% and 30% sucrose solutions, the samples were embedded in OCT^®^ compound (Thermo Fisher Scientific, Waltham, MA, USA), and cryosectioned at 10 µm thickness. Before staining, the sections were washed in PBS. To check the distribution of collagen, the slides were then immersed in Weigert’s Hematoxylin, Herovici’s working solution, and 1% acetic acid, respectively. Finally, the slides were dehydrated through a series of graded ethanol baths and cleared in a xylene bath. To visualize the expression of α-smooth muscle actin, sections were first blocked in 8% FBS buffer for 1 h, then incubated with α-smooth muscle actin monoclonal antibody (Thermo Fisher Scientific, Waltham, MA, USA, 14-9760-82) overnight at 4 °C. After washing with PBS 3 times, the slides were incubated with goat anti-mouse secondary antibody (Thermo Fisher Scientific, Waltham, MA, USA, A-11029). Sections were imaged using the BZ-X 810 digital microscope (Keyence, Osaka, Japan).

## 5. Conclusions

Wear debris polyethylene particles adversely affect the function of fibroblasts and increase the risk of fibrosis after joint arthroplasty. Here, we assessed the effect of cPE on the properties of fibroblasts, specifically their interaction with Mφs, and compared the resultant adverse cellular responses in MSC-Fs and iPSC-Fs in a 3D Mφ co-culture system. The properties of Mφs involved in the cPE-induced inflammatory process appear to be influenced by different sources of fibroblasts. Our results demonstrated that co-culture of MSC-Fs and iPSC-Fs with M2 Mφs in the presence of cPE activated the expression of fibroblast functional markers. However, co-culture of M1 Mφs with MSC-Fs resulted in an enhanced expression of fibroblast-related genes, while they remained at low levels when M1 Mφs were co-cultured with iPSC-Fs. Thus, we speculate that Mφs participating in the cPE induced inflammatory process are affected by the particles released from joint arthroplasty and the characteristics of fibroblasts.
